# AI-driven pathology in esophageal cancer: from early screening to precision prognostics

**DOI:** 10.3389/fonc.2026.1733548

**Published:** 2026-05-20

**Authors:** Yifan Bian, Jilei Li, Jiarui Cao, Sizhe Wang, Chunzheng Ma

**Affiliations:** 1 Henan University of Chinese Medicine, Zhengzhou, Henan, China; 2 Henan Province Hospital of Traditional Chinese Medicine (TCM), Zhengzhou, Henan, China

**Keywords:** artificial intelligence, digital pathology, esophageal cancer, precision oncology, prognostic prediction

## Abstract

Esophageal cancer (EC) represents a globally prevalent and highly aggressive malignancy, where early screening and precise diagnosis constitute foundational elements of contemporary therapeutic strategies, with pathologic review serving as the gold standard for definitive diagnosis and clinical assessment. This review comprehensively synthesizes recent advances in artificial intelligence (AI) models—leveraging diverse algorithmic frameworks—to augment pathological workflows across critical domains: early screening of Barrett’s esophagus (BE) and incipient EC; diagnostic refinement through invasion depth quantification, histopathological subtyping, and molecular pathology analysis; metastatic evaluation of lymph node involvement; prognostic prediction of patient survival; and efficacy assessment for multimodal therapies. These AI-driven methodologies demonstrate significant clinical utility throughout the EC disease continuum. We further critically examine persistent performance challenges and societal implications, offering insights to inspire future research toward precision oncology and optimized pathological efficiency.

## 1 Introduction

Esophageal cancer (EC) is one of the most common and devastating malignancies globally (1). It ranks as the sixth most frequently diagnosed cancer and the seventh leading cause of cancer-related mortality worldwide (2). The high mortality rate of EC stems partly from its insidious early symptoms and complex heterogeneity, which pose significant challenges for precise diagnosis and treatment (3).

Currently, the TNM staging system remains the cornerstone of clinical decision-making. However, its application hinges substantially on the subjective expertise of pathologists, leading to considerable inter-observer variability (4, 5). Take the assessment of submucosal invasion depth as an example: differences in assessors’ subjective experience and potential oversight of critical features can directly result in divergent conclusions (6). Therefore, this inherent error, stemming from reliance on subjective judgment, presents a compelling opportunity for computational pathology—namely, the development of objective, quantitative, and reproducible artificial intelligence (AI) tools (7).

**Figure 1:**
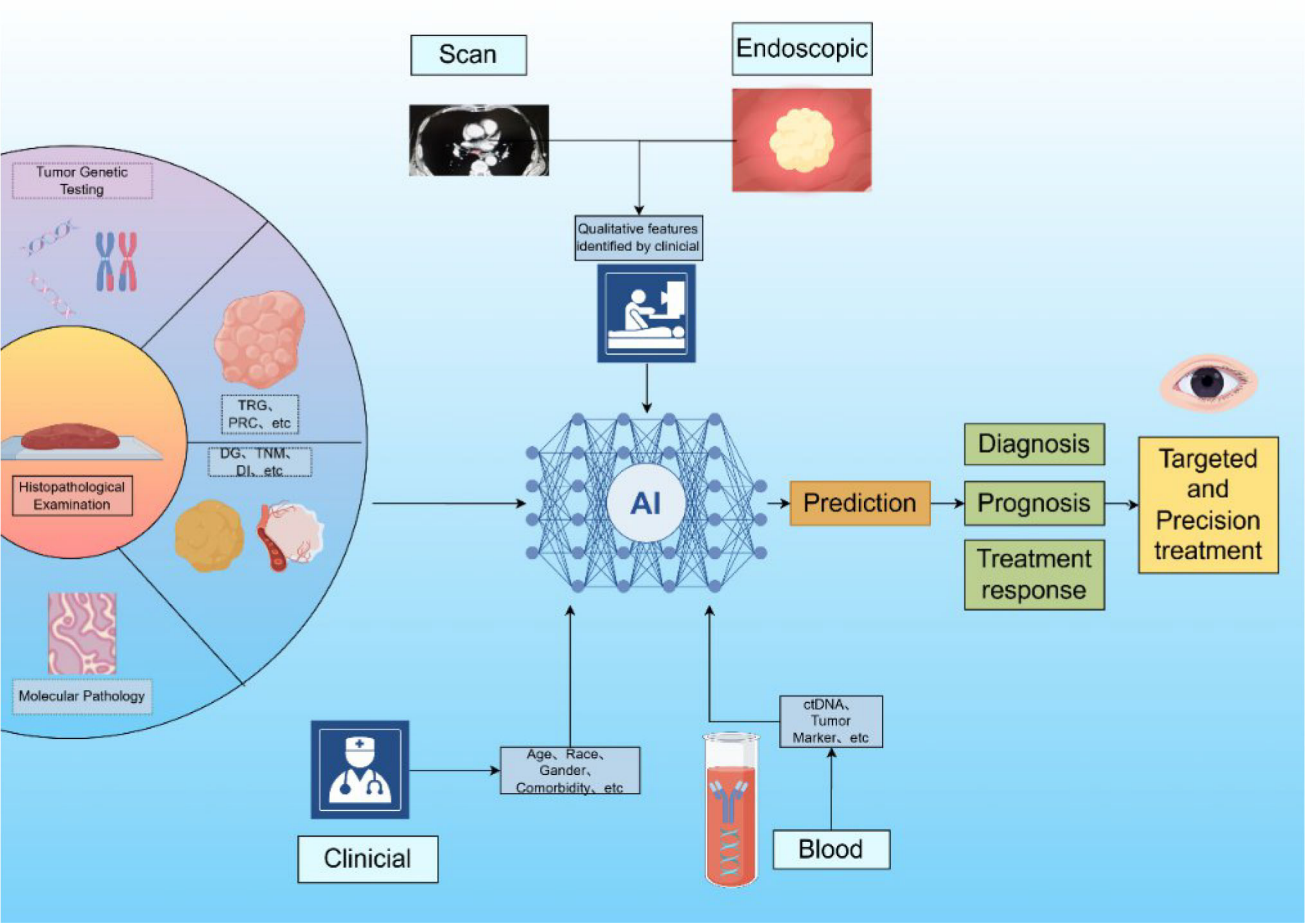
This figure illustrates AI’s workflow in pathologic diagnosis assistance: WSIs of biopsied tissue sections enables genetic testing, molecular pathology analysis, and evaluation of DG, TNM staging, DL features, TRG, and PRC. Integrated with clinically filtered endoscopic images, blood-based ctDNA/tumor marker profiles, and demographic/clinical data (age, ethnicity, sex, geographic location, comorbidities), AI provides key analytical support for patient diagnosis, prognosis assessment, treatment response prediction, and efficacy evaluation – thereby enabling precision targeted therapies.

Fortunately, remarkable progress has been achieved in the digitalization of pathological examinations (8). The widespread adoption of computer technology in pathology not only enhances imaging resolution through whole slide images (WSIs) and improves information-sharing efficiency, but also facilitates streamlined storage solutions (9) and establishes the technological groundwork for applying AI in pathology interpretation (10). AI exhibits significant potential for identifying subtle or complex features and specific pathological conditions traditionally challenging for human pathologists (11). This capability holds considerable importance for generating more accurate and comprehensive pathological reports, thereby informing crucial aspects of clinical management, including diagnosis, therapeutic decision-making, and prognosis assessment (**Figure 1**).

## 2 The architecture of current AI and the selection of multimodal fusion strategies

In the early stages of AI application in EC pathology, architectures like convolutional neural networks (CNNs), random forests (RFs), and U-Net laid the technical foundation for the field. Because of their strong hierarchical feature extraction capabilities, CNNs became the core architecture for image analysis tasks such as tumor identification and tissue classification (12). RFs build multiple decision trees and aggregate their predictions to handle nonlinear relationships in pathological feature spaces, serving as a primary model for prognostic prediction (13). U-Net and its variants achieve precise segmentation while maintaining spatial resolution (14), making them the mainstream choice for gland segmentation, tumor delineation, and invasion boundary detection. Additional methods like generative adversarial networks (GANs), gradient boosting machines (GBM), and extreme gradient boosting (XGBoost) were also widely used (15, 16). With their high training stability, ease of implementation, and strong cross-dataset generalization, these baseline models established the starting point and comparative benchmarks for AI research in EC pathology.

The spatial architecture of the tumor microenvironment (TME), particularly the distribution patterns of immune cells, has emerged as a key focus for prognostic analysis (17). Defining phenotypes such as “immune-excluded” or “immune-desert” has provided a novel biomarker system for evaluating immunotherapy responses and patient progression risks (18). However, because of the significant morphological heterogeneity and the complex of the TME in EC, traditional architectures show inherent limitations in integrating whole-slide-level correlations, modeling topological relationships, and aggregating multi-scale information (19).

To address the profound histological heterogeneity of EC and improve AI’s ability to analyze the TME, researchers are combining mathematically grounded and structurally novel architectures with traditional ones, or using them as functional alternatives. For instance, to capture the spatial structural heterogeneity of glandular architectures and invasion patterns, graph neural networks (GNNs) explicitly encode tissue as graphs of interacting cells or regions (20). At a more abstract level, GNNs combined with topological data analysis (TDA) can learn topological organization rules, providing noise-resistant descriptors (21). In parallel, vision transformers (ViTs) use self-attention mechanisms to integrate global contextual relationships across whole slides, improving the model’s understanding of diffuse infiltration and its ability to distinguish margins (22). To dissect cellular heterogeneity within the TME, spatial statistics combined with multi-task learning frameworks are being used to quantify the colocalization and interaction densities of different cell populations.

Although AI applications in pathology primarily rely on direct information from slides, incorporating multi-dimensional data—such as clinical information, genomics, and medical imaging—captures complex associations and complementary features. Consequently, multimodal fusion training has become a key component of current model development, outperforming the use of single modalities or simple feature concatenation. Regarding fusion strategies, approaches can be broadly divided into three types based on the level of data integration (23). Early fusion (EF) concatenates raw features to pre-model all interactions; while it requires higher architectural complexity, it achieves the maximum representation depth (24). Intermediate fusion (MF) uses separate encoders (e.g., CNNs for images and MLPs for omics data) to extract high-level features from each modality (25), which are then fused in the middle layers of the network. Because it balances flexibility and representation depth, MF has become the most mainstream strategy (26). Late fusion (LF) processes each modality separately and then integrates histopathological and molecular data at the decision layer, representing a relatively straightforward multimodal fusion method (27).

Current state-of-the-art models are no longer limited to the specific architectures mentioned above. Instead, they combine multiple architectures and algorithms to leverage their respective advantages, thereby refining their ability to extract and analyze information from pathology slides (28). By adopting multimodal fusion training to incorporate multi-dimensional data and using self-supervised learning (SSL) strategies to optimize the training process, these models achieve more complex functions, such as in-depth therapeutic efficacy analysis, prognostic prediction, and even survival assessment (29, 30).

## 3 Application of AI in precancerous lesion identification and early screening

Given the highly aggressive nature of EC, early screening is of paramount importance for enhancing patient prognosis (31). Although pathologic review serves as the definitive diagnostic gold standard, it encounters two principal challenges in clinical application: the precise detection of precancerous lesions and the accurate distinction between benign and malignant tissues. This is especially true for evaluating histological dysplasia and early carcinogenesis (32). The pathologic assessment of endoscopic biopsies forms the foundation of early screening initiatives, a process that demands both improved diagnostic consistency and streamlined workflow efficiency (33). To address these constraints, DL-based AI systems have recently surfaced as innovative tools. They offer potential by automating the recognition of histopathological features, quantifying biomarker expression levels, and facilitating the integration of multimodal data.

### 3.1 AI in pathological screening and diagnosis of Barrett’s esophagus

BE is a precancerous lesion characterized by the replacement of the distal esophageal stratified squamous epithelium with columnar epithelium (34) and is primarily caused by chronic gastroesophageal reflux disease (GERD). Pathologically, BE represents an adaptive metaplasia driven by mucosal response to acid and bile reflux (35). The cornerstone of its risk stratification lies in the identification of intestinal metaplasia and dysplasia, with high-grade dysplasia (HGD) carrying significant malignant potential (36). Approximately 90% of esophageal adenocarcinoma (EAC) cases arise from the progression of BE (37).

The model utilizes SSL pre-training to analyze H&E-stained slides, enabling precise identification of goblet cells and dysplastic glands without the need for manual annotation. By integrating quantitative assessment of immunohistochemical markers and employing a dual-model negative screening workflow where manual review is only triggered if either AI model detects positive immunohistochemical markers, this system reduces pathologists’ workload by 48% (38). This MF multimodal model also significantly improves the subtyping of intestinal metaplasia and the prediction of dysplasia progression risk, demonstrating strong discriminative performance (AUC = 0.85), and the integrated ViTs also provide reliable prognostic assessment compared to conventional histology alone (39). An MF model based on CNNs and GNNs with endoscopic images can simultaneously decode the spatial-topological correlations between endoscopic images and tissue sections. This achieves diagnostic consistency comparable to that of senior pathologists in identifying foci of intestinal metaplasia and early cancerous regions (40, 41). Furthermore, an AI system based on CNNs trained on large-scale datasets (containing over 100,000 samples) can assist in monitoring Barrett’s esophagus dysplasia and guide targeted biopsies, with sensitivity and specificity surpassing those of human physicians (Sensitivity: AI 96% vs. human average 79%; Specificity: AI 88% vs. human average 49%) (42).

Currently, the application of AI in predicting the longitudinal risk of EAC progression in Barrett’s esophagus patients remains in its early stages. Risk prediction focuses on interpreting the dynamic biological processes that signal malignant transformation, which requires models capable of integrating multi-timepoint pathological images to capture morphological evolution, along with genomic instability indicators and clinical risk factors (43). Emerging models use sequential DL frameworks to analyze changes in serial biopsy WSI sequences, and MF architectures can, to some extent, support reasonable inference (44). However, due to the limited stability of current models in processing multidimensional medical data, their development relies on large-scale, longitudinal, multicenter cohorts and temporally consistent pathological samples, which substantially increases the computational and financial costs.

### 3.2 AI in pathological screening and histological diagnosis of early EC

Early EC screening constitutes a cornerstone strategy for improving patient prognosis, demonstrating particularly significant clinical value in early-stage disease—curative-intent treatment can elevate five-year survival rates beyond 90% (45, 46).

The model based on ViTs enhances the processing of high-resolution pathological images through an image patching strategy, thereby improving the detection of early-stage EC and its subtype classification. These systems achieve superior performance (AUC = 0.93) compared to assessments by pathologists (AUC = 0.85) (47). In patients with superficial EC, models that assess the depth of invasion can differentiate between shallow and deep submucosal infiltration via subpixel-level boundary delineation, offering key decision support for selecting indications for endoscopic submucosal dissection (48).

Furthermore, a study utilizing an AI system incorporating GNN models compared the performance of CNN and ViT in analyzing gastrointestinal endoscopic images, finding that ViTs demonstrated better generalization and robustness in dynamic clinical environments (49). Serum biomarker models based on GBM have optimized the specificity of screening for esophageal squamous cell carcinoma (ESCC) by detecting super-enhancer-driven secretory proteins (50). Constrained attention multiple instance learning (CAML) models can identify key histopathological markers of malignant transformation by quantifying vascular density and morphological abnormalities in invasive regions (44). An MF multimodal model, which combines endoscopic and pathological data, achieves diagnostic concordance comparable to that of experienced pathologists (51). CNNs enable accurate identification of precancerous lesions and early carcinomas by analyzing endoscopic images and extracting temporal video features (52). Such models also assist cytodiagnosis systems in accurately discriminating early carcinoma from high-grade intraepithelial neoplasia (HGIN) using low-grade squamous intraepithelial lesion (LSIL) as a diagnostic threshold; their translational potential for large-scale population screening has been validated experimentally (53).

However, studies have also shown that real-time AI assistance does not significantly improve the detection rate of ESCC by non-specialist endoscopists when sufficient training data are lacking (54). Moreover, AI systems trained exclusively on high-quality images may perform poorly in heterogeneous clinical settings; their robustness can be enhanced through reinforced training strategies and scaled-up training datasets (55).

## 4 Application of AI in auxiliary staging of EC

Accurate pathological staging is crucial for prognosis. However, determining the deepest front of tumor invasion (T staging) and screening lymph nodes for micrometastases (N staging) are not only labor-intensive but also highly challenging due to histological ambiguity and the difficulty of detecting subtle lesions (56). To overcome these quantification challenges, AI-driven tools have been introduced into the diagnostic workflow. For instance, semantic segmentation models are utilized to precisely delineate tumor-stroma boundaries, and high-sensitivity detection algorithms are employed to identify micrometastases within complex lymphoid tissue backgrounds. These approaches assist pathologists in making staging decisions more accurately and efficiently (57, 58).

### 4.1 Application of AI in pathological detection of invasion depth

Pathologically, the invasion depth of EC is defined as the extent of tumor penetration through the epithelial basement membrane into deeper esophageal wall layers (59). Accurate assessment of this parameter relies on histopathological examination, combined with endoscopic ultrasonography (EUS) and magnifying endoscopy, to identify submucosal layers (SM1-SM3) (60). As a critical determinant for clinical treatment decisions, this parameter shows a significant positive correlation with lymph node metastasis rates and serves as a major predictor of five-year survival (61).

Models based on deep neural networks (DNNs) and those incorporating GNNs can optimize tumor mutational burden (TMB) prediction by extracting immunohistochemical features from pathological specimens, which significantly improves molecular subtyping efficiency (Pearson r = 0.98/0.82/0.92) (62). Such systems also achieve expert-level accuracy in differentiating mucosal layers from SM1 in superficial ESCC (63). Furthermore, LF multimodal models can accurately evaluate key pathological indicators, including lymphatic invasion, vascular invasion, and tumor invasion depth (64). Models based on the U-Net framework that incorporate multidimensional data for boundary delineation show agreement with expert classifications in SM staging. They achieve automated invasion quantification with a high intersection over union (IoU) on the primary dataset (mucosa: 93.81%; tumor: 91.95%). However, performance on external validation sets dropped to 59.86% and 50.88%, highlighting the need for improved robustness (65). This indicates that analyzing the spatial topological structures in pathological slides can significantly reduce measurement errors in invasion depth.

### 4.2 Application of AI in pathological detection of lymph node metastasis

In EC, assessing lymph node metastasis (LNM) primarily focuses on quantifying the extent of malignant cell spread within regional lymph nodes (66, 67). Given the strong negative correlation between metastatic burden and patient prognosis, the precise identification of occult micrometastases is crucial for prognostic prediction and treatment planning. Consequently, LNM quantification serves not only as a primary indicator for adjuvant therapy but also as a dynamic biomarker for adjusting the intensity of personalized treatment (68).

An MF multimodal imaging system incorporating endoscopic images has achieved sub-millimeter identification of ESCC metastasis, demonstrating diagnostic performance comparable to that of senior pathologists in determining LNM (44). By integrating TDA, such approaches can construct preoperative LNM risk prediction frameworks, significantly improving the accuracy of clinical staging (69). Regarding recurrent laryngeal nerve lymph nodes, DL models can predict metastasis risk based on baseline pathological features, providing key evidence for determining intraoperative dissection boundaries, formulating postoperative treatment plans, and evaluating prognosis (70). Similarly, DL models combined with TDA can extract short-axis morphological features and spatial topology information from lymph nodes to optimize preoperative quantification of metastasis risk (71). By analyzing micrometastases in radical resection specimens at sub-pixel resolution to evaluate spatial topology, these methods maintain high diagnostic sensitivity even in complex pathological environments (72).

### 4.3 Application of AI in metastatic risk assessment and tumor origin tracing for EC

Quantification of metastatic risk also partially influences the selection of treatment regimens (73). High-risk patients require intensified systemic therapies, including PD-1/PD-L1 inhibitors combined with chemotherapy, whereas patients with specific metastatic patterns, such as liver or lung metastases, may be candidates for surgical intervention (74). Patients presenting with osseous or cerebral metastases require radiotherapy or targeted interventions. Tracing the origin of metastasis by interpreting the molecular features of the primary tumor can help adjust precision treatment and represents a key pathway for improving patient prognosis (75).

An MF multimodal model has demonstrated high reliability in tracing the origin of primary tumors by analyzing key indicators, including methylation haplotypes, copy number variation features, CpG island hypermethylation, and chromosome instability-driven deletions. This model achieved robust cross-center validation in cases of carcinoma of unknown primary (CUP), attaining an accuracy of 99% in internal validation and 93% in independent external testing (76). A microRNA biomarker analysis system based on an RF achieved cell lineage tracing in metastatic lesions through spatial transcriptomic analysis of tumor-specific expression features directly from tissue touch imprints (77). A model combining CNNs and ViTs integrated 11 TME indicators to construct a dynamic prediction model for liver metastasis risk (78). By using GANs to synthesize simulated images of liver microvascular invasion, the system significantly enhanced the early detection capability for minute lesions (<5 mm). Furthermore, such systems can quantify risk for lung metastasis by extracting radiomic features via 3D convolutional kernels (e.g., primary-to-hilar lymphatic connectivity and subsolid nodule growth rate) and integrating multidimensional data such as histopathological subtypes and staging information (79).

The above cases demonstrate that current AI models are capable of identifying micrometastases and subtle invasive growth patterns. However, the clinical utility of these advancements remains controversial, limited by the risk of false negatives in biopsy samples and because prognostic assessment of distant metastasis primarily relies on the presence or absence of lesions rather than the precise quantification of metastatic burden (80).

## 5 AI in histopathological diagnosis

The pathological classification of EC, achieved by integrating morphological and molecular features, is a crucial part of pathological diagnosis and directly determines the formulation of treatment plans (81). For instance, ESCC exhibits high radiosensitivity and is suitable for concurrent chemoradiotherapy; in contrast, EAC necessitates combined targeted therapy or immune checkpoint inhibitors (82, 83). Furthermore, analyzing molecular pathological features to quantify metastatic potential and therapeutic responsiveness represents a promising future direction for AI in this field (84).

An EF multimodal image fusion system achieves bidirectional conversion between three-dimensional and two-dimensional pathological information, enhancing the sensitivity of micrometastasis detection through spatial topological analysis (85). In the evaluation of tumor regression grading (TRG) for surgical specimens, a CNN modal achieves diagnostic concordance comparable to that of senior pathologists, significantly improving inter-observer agreement and diagnostic efficiency in identifying histological substructures (86). Through quantitative glandular structure analysis, such architectures can automate the discrimination between low-grade and high-grade intraepithelial neoplasia, thereby substantially reducing unnecessary workload (87). Additionally, an integrated diagnostic platform driven by RF overcomes the limitations of detecting low-abundance circulating proteins by fusing serum biomarkers with clinical data, significantly optimizing the screening efficacy for early-stage ESCC (88).

## 6 AI-assisted pathological detection for prognostic prediction

Clinical decision-making for EC requires the integration of multifactorial clinical parameters to predict treatment outcomes and risks, enabling individualized management (Yang et al., 2023). However, disease heterogeneity and host biological variability limit the efficacy of current prediction models for multimodal therapies (surgery, neoadjuvant therapy (NAT), and immunotherapy). This presents significant challenges, particularly in optimizing therapeutic windows by balancing toxicity with survival benefits (89, 90). Recently, AI has made progress in analyzing multidimensional medical data, enabling the quantitative evaluation of the efficacy and safety profiles of patient-specific regimens, making the prediction of treatment response possible.

### 6.1 Application of AI in patient prognosis prediction

Although the TNM staging system and histological classification form the foundation of current prognostic assessment, their predictive efficacy is significantly limited by individual patient differences, such as performance status, age, comorbidities, and nutritional status. Accurate prognostic analysis not only helps prepare patients with expected poor outcomes psychologically but also reduces anxiety in those with favorable prognoses, ultimately improving their overall quality of life (91). However, determining the prognosis of EC involves multidimensional and complex variables, which still carries a considerable risk of prediction errors (Yang et al., 2023).

Multiple studies have confirmed that AI shows great potential in improving prediction accuracy by integrating multi-source heterogeneous data and quantifying microscopic features. For instance, a DL-based desmoplastic reaction (DR) classification model (92) demonstrated high accuracy in prognostic prediction, outperforming manual evaluation in both reliability and efficiency (Dice = 0.81, indicating high spatial agreement). Abnormal cancer/testis antigen (CTA) expression leads to poor prognosis by driving aberrant proliferation and remodeling the immune microenvironment. To address this, a multimodal model based on the Stable Diffusion architecture has been used to examine the association between CTA protein coding and clinical features, achieving superior predictive performance (93). Similarly, a DL multimodal model that fuses pathological, radiomic, and clinical data can simultaneously predict programmed death-ligand 1 (PD-L1) expression, immunotherapy response, and overall survival, showing efficacy comparable to that of experienced pathologists (94).

A CNN-driven circulating tumor cell (CTC) analysis platform (95) predicted prognosis, metastasis risk, and treatment response with high precision and reduced time. Because it operates independently of known biomarkers, this platform highlights its clinical potential in ESCC. By integrating somatic mutation data with pathological images, a DNN model (62) showed robust predictive capability. It can accurately quantify TMB and generate prognostic scores for EC, and provides a visually interpretable framework for outcome prediction. An RF model focusing on immune cell infiltration, functional states, and immune factor expression was used to construct overall survival and prognostic scoring systems (96), offering significant value for formulating personalized immunotherapy strategies. Additionally, a DL model dedicated to pathological sections (97) achieved reliable prognostic assessment via precise nuclear counting and Ki-67 quantification. Finally, an SSL framework combining RF, XGBoost, and multivariate Cox proportional-hazards regression (COX) predicted prognosis by identifying the differential expression of mitophagy-related genes. This not only validated the critical role of this pathway in prognostic evaluation but also achieved risk stratification and guided adjuvant chemotherapy decisions (98, 99).

### 6.2 AI in radiotherapeutic efficacy assessment and complication prediction

Optimizing EC radiotherapy requires a comprehensive evaluation of disease status and patient physiology. Accurate prediction of treatment efficacy, therapy-related toxicities (e.g., radiation pneumonitis, esophagitis), and severe complications is essential for risk-benefit evaluation and treatment individualization (100). Current experience-based decision-making, coupled with unforeseen individual risk factors, increases clinical uncertainty (7). To help clinicians overcome these limitations, AI-driven predictive models are utilized to integrate multidimensional data.

For instance, a DL model demonstrated robust performance in predicting grade 4 radiation-induced lymphopenia, achieving an AUC of 0.831, an accuracy of 0.769, an F1-score of 0.631, a precision of 0.670, and a recall of 0.610 (101). In another study, an evaluation framework established by assessing and quantifying lactate metabolism biomarkers achieved therapeutic response prediction accuracy that surpassed conventional methods (102). Similarly, CNNs trained on pre- and intra-treatment clinical-pathological data from EC patients receiving proton or photon radiotherapy can provide robust risk stratification for severe myelosuppression, even in the presence of patient heterogeneity. The extensible design of these CNN models also facilitates the investigation of various treatment-related adverse events (103). Additionally, CNNs can analyze radiation-induced morphological changes to quantify local tumor response dynamics. This approach provides an objective assessment of both immediate effects and long-term trajectories for adaptive treatment adjustments (104).

### 6.3 AI in prediction of surgical complications

Surgical resection remains the cornerstone of curative-intent therapy for EC. By rapidly removing the primary tumor and performing regional lymphadenectomy, it offers the highest probability of cure, reduces metastatic risk, and provides a basis for definitive pathological diagnosis and staging (105, 106). Nevertheless, this major intervention carries significant physiological and immunological burdens, making it challenging to accurately weigh surgical benefits against potential harms for optimal decision-making (107, 108).

In this context, AI offers valuable tools. Models based on ViTs can preoperatively predict the efficacy of NAT in ESCC. Trained on large-scale datasets, these models exhibit high efficiency and robustness, facilitating personalized treatment strategies (109). Furthermore, by analyzing computational pathology features within the TME, CNNs have shown the ability to predict EC prognosis, such as by evaluating 90-day postoperative mortality risk (110, 111). While the discriminative ability of these models still requires validation in larger cohorts before broad clinical translation, the current findings represent a meaningful step forward in advancing personalized patient management and enhancing surgical safety.

### 6.4 AI in predicting immunotherapeutic efficacy and complications

Immunotherapy has significantly transformed cancer management by activating the patient’s immune system against tumors. It effectively inhibits disease progression and reduces tumor burden (112, 113). However, its clinical application is limited by off-target toxicities that damage normal tissues and a reliance on the expression of specific immune targets, such as PD-L1. While evaluating existing biomarkers through immunohistochemistry or genetic testing allows for a preliminary benefit assessment, there remains significant room to improve prediction accuracy using AI (108).

MF multimodal models combine genomic and transcriptomic profiles with histopathological images to predict patient sensitivity to immunotherapy and related clinical outcomes, thereby optimizing treatment selection (114). These models can also directly quantify HER2 expression, spatial distribution, and cell density within EC tissues. This approach establishes significant correlations between histomorphological features and immunotherapy efficacy, providing a basis for pretreatment prediction (115). RF helps identify and prioritize novel targets, such as neoantigens, from pathological specimens. This improves the reliability of neoantigen prediction to facilitate clinical translation (116). Furthermore, GBM models can uncover the underlying mechanisms of immunotherapy response from tissue sections, demonstrating strong prognostic capabilities even with limited sample sizes (117). Compared to manual interpretation, CNN-based pipelines enhance the objectivity, speed, and standardization of pathology workflows by automating the quantification and segmentation of immunohistochemical markers (118). For instance, CNN models have extracted radiomic features from the pathological specimens of ESCC patients treated with PD-L1 inhibitors, enabling an efficient assessment of treatment efficacy and prognosis (119).

### 6.5 AI in predicting efficacy and complications of neoadjuvant chemotherapy

NAT is the standard preoperative systemic treatment for locally advanced EC. It downstages tumors and provides a critical window to assess treatment sensitivity (120, 121). Pathological complete response (pCR) is the primary indicator of efficacy, and patients achieving pCR have significantly better survival outcomes than those who do not.

AI establishes a histology-based efficacy prediction paradigm. CNNs extract multi-scale morphological features from tumor regions to generate predictive profiles for treatment response (122). Furthermore, multimodal frameworks that integrate spatial metabolomic profiles of EAC tissues can quantitatively analyze metabolic heterogeneity between epithelial and stromal compartments, thereby improving prediction accuracy (123). These models successfully predict neoadjuvant chemotherapy response. Moreover, their ability to assess metabolic responses provides unique value for prognostic stratification.

## 7 Application of AI in microbiota detection of EC tissues

As key components of the TME, microbial communities affect disease progression through multiple mechanisms, including immune regulation and nutrient metabolism (124, 125). Certain commensal bacteria can trigger anti-tumor immune responses to inhibit carcinogenesis. Conversely, the overgrowth of opportunistic pathogens can aggravate local inflammation and tissue damage, which increases the risk of secondary infections and hemorrhage, ultimately leading to a poorer prognosis (126).

AI provides a new approach to understanding these complex microbe-host interactions. For instance, an MF multimodal model based on the LightGBM algorithm (127) can efficiently identify the heterogeneity of microbial composition within EC tissues. By integrating multidimensional features, this model accurately quantifies microbial abundance and community structure. This lays the technical groundwork for discovering microbiome-related biomarkers and developing clinical detection tools.

## 8 Summary and discussion

AI applications in tumor pathology have emerged as a global research focus (128). Its core advantage initially lay in the automated detection of mitotic figures using image recognition (129). As algorithms and hardware continue to improve, AI has expanded into multidimensional pathological analysis. It is being continuously optimized in traditional areas such as image segmentation, pathological subtype classification, and microsatellite instability status evaluation. At the same time, it is making gradual progress in handling complex tasks, including fluorescence image interpretation and spatial transcriptomic data integration (130). Furthermore, certain systems combine generative models with logical reasoning engines, enabling AI to directly extract quantitative features from pathological images and generate diagnostic conclusions through multimodal data fusion (131). Finally, the widespread use of AI in CT and MRI examinations has laid a solid foundation for its clinical deployment in pathology. This prior implementation experience includes infrastructure setup and staff training protocols, which significantly pave the way for AI to be practically integrated into routine clinical workflows.

**Figure 2:**
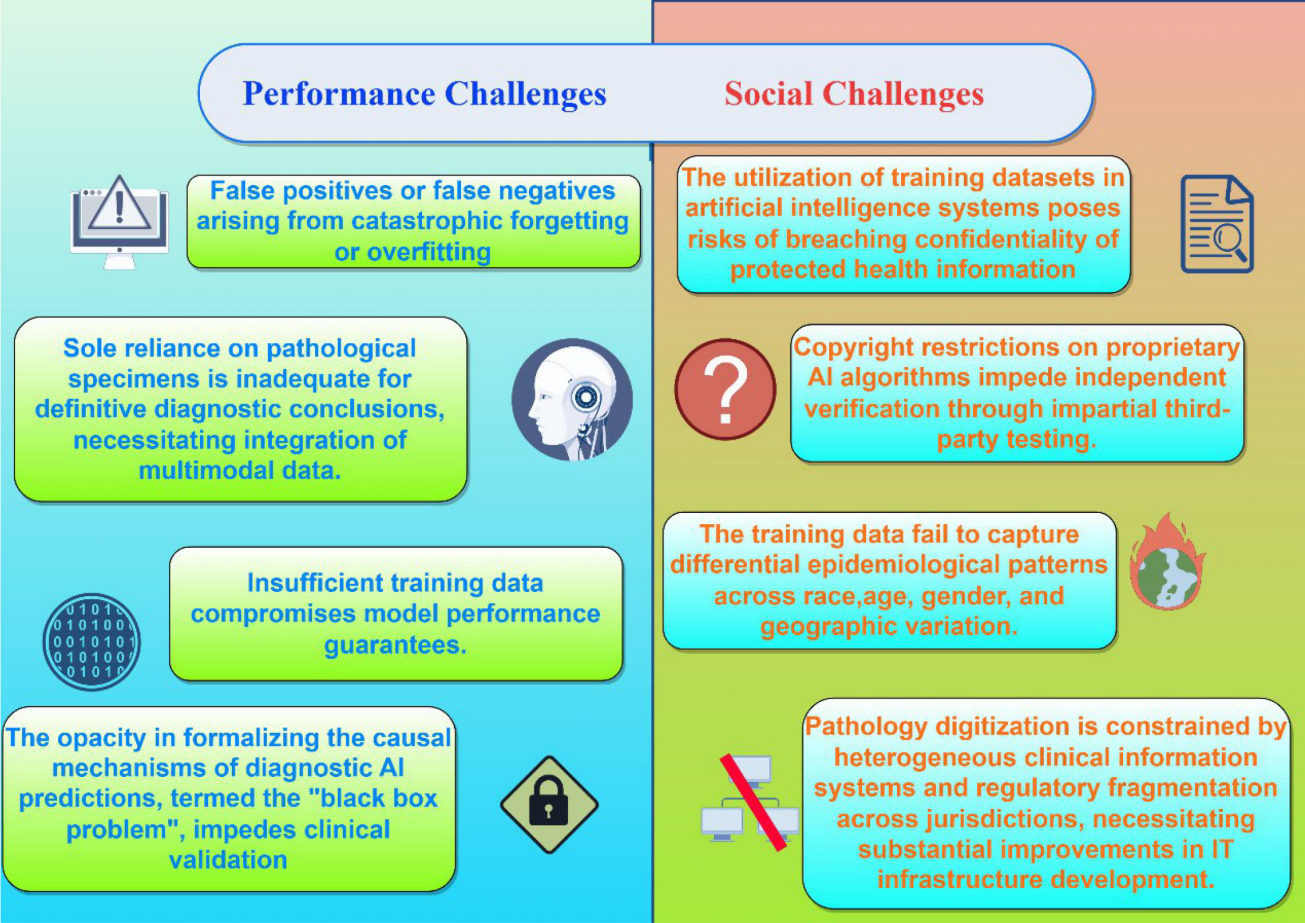
Challenges in AI-assisted pathology: Technical limitations of model performance (left) versus broader societal barriers in development/implementation (right).

This review systematically summarizes three core values of AI in EC pathology: enhancing diagnostic precision and efficacy, and identifying novel therapeutic targets. Current studies confirm that AI’s potential in this field may reshape clinical pathology workflows. However, its comprehensive implementation requires overcoming key technical and ethical bottlenecks (**Figure 2**).

### 8.1 Technical challenges in AI model performance

First, although most AI models demonstrate diagnostic efficacy comparable to or surpassing senior pathologist teams in validation studies, unresolved issues persist regarding DL’s inherent catastrophic forgetting (where new knowledge overwrites prior learning during continuous training) and overfitting (excessive sensitivity to noise in training datasets) (132, 133). These challenges are particularly acute in EC pathology due to the disease’s histological heterogeneity and molecular subtype diversity, which severely test models’ generalization capabilities.

Second, current models require multimodal inputs—including endoscopic images, CT/MRI scans, and clinical records—to achieve optimal performance, whereas human pathologists attain high diagnostic precision using histopathological slides alone (134, 135). Although AI significantly outperforms humans in processing multimodal data efficiently, the substantial difficulty of acquiring complete multimodal datasets in clinical practice critically limits real-world utility.

Third, successful commercial medical imaging AI models typically require millions of training samples, yet existing pathology AI models generally train on merely thousands of cases, with some datasets containing fewer than 100 specimens (136, 137). While sophisticated algorithm design can achieve promising results with limited samples, insufficient data scale inevitably compromises model stability in real-world scenarios. Crucially, most datasets lack epidemiological representativeness: EC incidence varies significantly by race, geography, and comorbidities, but these variables are rarely incorporated during training, causing performance degradation in cross-population applications (138, 139). Such data bias may increase missed diagnoses in early screening contexts.

The need to develop separate algorithmic approaches for ESCC and EAC—two etiologically distinct subtypes of EC—considerably complicates model construction. Compounding this challenge, marked geographical disparities in subtype prevalence indicate that models trained on regionally imbalanced datasets may exhibit poor global generalizability, thereby risking amplification of healthcare disparities (140).

Finally, the “black-box” nature of DL decision-making impedes clinical adoption. Although AI can visualize regions of interest, the rationale underlying its cell-level discrimination remains uninterpretable. This not only hinders algorithm optimization but also erodes clinicians’ confidence in AI conclusions (141).

Furthermore, the promotion of AI in tumor pathology faces not only technical constraints but also complex socio-ethical challenges:

### 8.2 Social challenges in AI implementation

First, despite AI’s emergence as a critical medical tool, its training relies on massive clinical datasets containing sensitive patient information (142). Particularly in high-stakes studies like EC, balancing data scarcity with privacy protection remains contentious—a fundamental reason for inadequate training data in many models.

Second, current AI training depends heavily on decentralized electronic health records (EHRs) (143). Although national-level data governance frameworks exist, incompatible architectures and interface standards across institutions necessitate costly preprocessing for cross-center data fusion, further limiting access to high-quality training datasets.

Third, despite partial disclosure of algorithmic frameworks, internal feature weighting mechanisms and decision pathways remain opaque due to commercial confidentiality or customization needs (144). This lack of traceability severely impedes clinical trust in pathology contexts requiring explicit diagnostic evidence.

Finally, most studies rely on developer-curated test sets for performance validation, while independent third-party replication remains scarce. Although limited verification attempts exist for published models (145), systematic large-scale external validation is unfeasible due to intellectual property restrictions and patient privacy clauses—casting doubt on real-world efficacy.

Addressing these multidimensional challenges requires a dual-pathway approach:

### 8.3 Future directions and required efforts

Technologically, core efforts should focus on continuous algorithmic innovation: Developing anti-forgetting incremental learning mechanisms and regularization strategies can effectively mitigate overfitting and catastrophic forgetting in DL, thereby reducing models’ excessive reliance on massive training datasets. Explainability research is key to addressing the “black box” dilemma: the field is evolving from general techniques like feature visualization toward domain-specific explainable AI. Methods such as concept activation vectors and counterfactual analysis link model decisions to histopathologically meaningful concepts, providing interpretable, clinically actionable insights essential for integrating AI into verifiable diagnostic workflows.

Addressing catastrophic forgetting in digital pathology demands specialized incremental learning strategies, particularly given the unique scale and prohibitive costs associated with annotating WSIs. Unlike natural images, WSIs present a continual stream of high-resolution patches alongside rare morphological patterns that complicate standard learning approaches. Effective strategies might leverage a combination of replay-based methods that store representative patches from prior tasks, combined with prompt-based tuning of large foundation models. Such an approach enables the integration of new cancer subtypes or staining protocols without necessitating retraining from scratch or compromising performance on previously learned diagnostic criteria. The application of SSL strategies has also demonstrated considerable promise in reducing training costs and complexity while substantially improving model efficacy.

Data governance frameworks must strike a delicate balance between ethical considerations and practical efficacy. To address the root cause of training data scarcity, secure sharing mechanisms grounded in tiered authorization and differential privacy should be established. Government-led initiatives promoting cross-institutional standardization of medical data would facilitate the efficient, compliant integration of multi-source pathological information. Verification systems urgently require third-party involvement: while safeguarding intellectual property and patient rights, open scientific auditing platforms should be developed to allow independent teams to reproduce model performance and trace training data provenance. Such an approach would help identify potential biases while simultaneously fostering greater acceptance of AI within the medical community.

Data strategies must strengthen epidemiological orientation: Prioritize collecting multidimensional data from high-risk populations across diverse ethnicities, regions, and baseline disease states, ensuring biological representativeness and clinical generalizability of training sets to optimize model performance in real-world scenarios. While AI demonstrates the potential to standardize pathological workflows, its real-world implementation requires addressing data heterogeneity and model interpretability barriers.

### 8.4 Challenges in the development of novel models

Novel model architectures, such as GNNs and ViTs, are universally characterized by high complexity, difficult implementation, and substantial computational demands. They also require massive amounts of initial training data to converge sufficiently before proceeding to the SSL phase. These factors collectively restrict their potential for development and practical application. Furthermore, the self-attention mechanism of ViTs renders them inefficient when processing high-resolution pathological images (48). They must rely on complex patching strategies, which inadvertently disrupt the overall continuity of the tissue. Although TDA offers robust mathematical capabilities, translating its highly abstract descriptors into morphological features that pathologists can intuitively understand introduces an additional cognitive barrier or computational cost (146). Ultimately, cost-related issues remain the primary obstacle in the development of such models.

As current state-of-the-art models utilize SSL objectives to pre-train on massive amounts of unlabeled WSIs, this paradigm shift enables the construction of versatile models for tasks ranging from subtype classification to prognostic prediction. It also improves compatibility across different institutions and staining protocols, partially reducing the annotation scale and costs required by traditional models (147). However, a semantic gap exists between SSL pre-training tasks and downstream target tasks, meaning the general representations acquired during pre-training cannot be directly applied to specific medical tasks. Due to the lack of unified and efficient evaluation standards for assessing the inherent representation quality of pre-trained models, their performance relies heavily on the results of downstream fine-tuning. MF strategies face analogous challenges (148). Although intermediate fusion is flexible, the design of its network architecture and the selection of the optimal fusion point still lack theoretical guidance and unified standards, leading to significant variability in model performance.

### 8.5 The biological significance and future development of model architecture

To address the limitations of existing models in capturing long-range spatial dependencies and complex tissue ecosystems, AI is shifting toward domain-specific innovations tailored to the unique biological complexities of the disease. This shift has prompted a growing interest in designing biologically inspired models to elucidate the mechanisms of disease progression. This progress signals a broader paradigm shift. Instead of merely serving as a tool for task-specific automation, AI is evolving into an integrated engine for discovery and precision oncology. Ultimately, it may even act as a consultative assistant to support physicians in clinical decision-making (149).

The next frontier will likely be defined by large-scale, multimodal foundation models. These models will be pre-trained on massive datasets that combine histopathology images with genomic and clinical data. By integrating explainable AI techniques, such as concept activation and counterfactual analysis, these systems can reveal subvisual prognostic features and generate novel biological hypotheses (such as 98). Realizing this potential requires overcoming several challenges, including data standardization, multimodal fusion, and robust clinical validation. However, achieving these goals promises to usher EC care into a new era of data-driven, personalized management.

## 9 Conclusion

In summary, this review synthesizes applications of AI in early diagnosis, screening, histopathological classification, and prognostic and therapeutic response assessment for EC. AI demonstrates substantial potential to address issues of objectivity and inter-observer variability in histopathology while significantly enhancing pathologists’ workflow efficiency. It underscores the critical importance of ongoing algorithm refinement to facilitate seamless integration into routine clinical workflows for EC patients.
